# A Multifunctional Aggregation-Induced Emission Luminogen with pH-Response Detachable Connector for Lipid Droplet-Specific Imaging and Tracing

**DOI:** 10.3390/molecules28207029

**Published:** 2023-10-11

**Authors:** Yanjie Li, Rui Fan, Pengfei Gao, Chang-Hua Hu

**Affiliations:** 1 College of Pharmaceutical Sciences, Southwest University, Chongqing 400715, China; lyj1989@swu.edu.cn; 2 Southwest University Hospital, Southwest University, Chongqing 400715, China; fanrui01@swu.edu.cn; 3 Key Laboratory of Luminescence Analysis and Molecular Sensing (Southwest University), Ministry of Education, Southwest University, Chongqing 400715, China; 4 NMPA Key Laboratory for Quality Monitoring of Narcotic Drugs and Psychotropic Substance, Chongqing 401121, China

**Keywords:** aggregation-induced emission, AIEgens, lipid droplets, acid response, lysosome

## Abstract

Lipid droplets (LDs) targeting probes are important for investigating the biological functions of LDs. The interplay between LDs and some other organelles can help to further understand the biological functions of these organelles. However, it is still a challenge to design functional probes that can specifically target LDs and are responsive to some other organelles. Herein, a multifunctional aggregation-induced emission luminogen (AIEgen), namely the TPA-CN, was prepared by the simple aldimine condensation reaction for lipid droplet-specific imaging and tracing. TPA-CN can be sensitively responsive to the acid environment of lysosomes due to the pH-response detachable connector in TPA-CN. With the assistance of this characteristic, it can be concluded from the fluorescence imaging and co-localization analysis results that the internalization of TPA-CN and the targeting of LDs does not involve the lysosome and the lysosomal escape process. At last, the TPA-CN was successfully used for the high-sensitivity imaging of dynamic information of LDs.

## 1. Introduction

Lipid droplets (LDs), as the most important reservoirs of neutral lipids in cells, are indispensable for energy storage and many energy-demanding processes. In addition, LDs are also associated with several diseases, such as diabetes, obesity, and cancer [1]. Therefore, LD-targeting imaging is urgently needed for investigating the biological functions of LDs, and thus, the related fluorescent probes have received widespread attention. As known, aggregation-induced emission luminogens (AIEgens) always demonstrate incomparable advantages in bio-imaging [2,3,4,5,6,7,8,9,10,11,12]. As a result, AIEgen-based LD-targeting imaging has played important roles in the recognition and understanding of LDs [13,14,15,16,17]. During this process, LD-targeting AIEgens with specific capabilities are developed, such as those with near-infrared wavelength [18,19], photoactivatable ones [20], those with a large two-photon absorption cross-section [21,22], those with ultralow used concentration [23], and so on. In addition, LD-specific imaging has also been extended to the research of bacteria and green algae [24,25].

To better understand the biological functions of organelles, the investigation of the interplay between organelles, including the related material and energy transfer, is important. Actually, all the organelles, including LDs, are not isolated in terms of structure or function. For example, the coordinated autophagic degradation of LDs with mitochondria has been confirmed as a route of hepatic steatosis in the process of hepatocellular carcinoma [26]. A single AIEgen bearing lipophilic, cationic, and hydrolyzable moieties was successfully used to evaluate cell viability based on its distinct two-color emission changes and targeting imaging of mitochondria and LDs, respectively [27]. In order to investigate the interplay between organelles, functional probes that can be used to target and illuminate two or more organelles are needed.

Up to now, based on functional AIEgens, the interplay between LDs and some other organelles has been explored. Importantly, the stimulus-responsive AIEgens might be helpful to investigate the positioning. With the assistance of the single fluorescent probes capable of simultaneous and two-color imaging of LDs and the endoplasmic reticulum (ER), valuable proof for the mainstream hypothesis that LDs originate from the ER was supplied [28]. The interplay of lipid droplets (LDs) and lysosomes plays an important role in cell metabolism, and the visualization of this process can provide useful information on organelle communication and function. In addition, many dyes and probes have been reported to be internalized by cells through lysosomes and further distributed after the lysosomal escape process. By using a functional AIEgen that localizes in lysosomes and LDs with red and cyan emissions, the dynamic process of this probe distributed in these two organelles and the consumption of LDs can be visualized [29]. However, functional probes for investigating the LD–lysosome-related bioprocess are still rare.

**Scheme 1:**
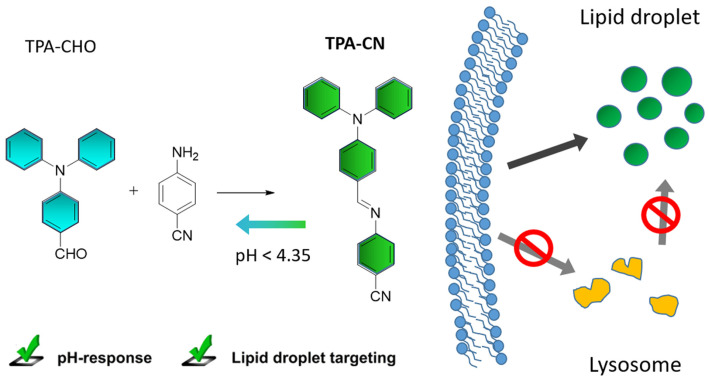
Schematic representation of the acid-responsive property of triphenylamine-benzonitrile Schiff base (TPA-CN) molecule and its intracellular pathway imaging analysis.

Therefore, developing functional probes that can record and judge their transfer path from the internalization to the LDs is still critical. As known, the lysosome always exhibits an acidic environment [30], and the pH is mainly in the range of 4–5 [31,32,33]. Accordingly, acid-responsive AIEgens might be helpful in exploring the lysosome-related biological process [34,35,36,37]. Herein, we designed an AIEgen, TPA-CN, which can target LDs and respond to the acidic environment of the lysosome based on the acid-responsive imine bond. Based on these characteristics, it can be concluded that the internalization of TPA-CN and the targeting of LDs do not involve lysosomes and the lysosomal escape process (Scheme 1).

## 2. Results

### 2.1. Synthesis and Characterization of TPA-CN

In order to investigate whether the internalization process of the LD-targeting probe passes through the lysosome, we designed a functional AIEgen that can target the LDs together with the acid response property. Based on the simple aldimine condensation reaction, the fluorescence wavelength of TPA-CHO could be red-shifted to a longer wavelength due to the introduction of the electroactive cyan group. In addition, the acid response of the imine bond was selected as a pH-response detachable connector. The synthetic procedure of the triphenylamine-benzonitrile Schiff base (TPA-CN) is shown in Figure S1. The ^1^H and ^13^C NMR spectra and the high-resolution mass spectra (HRMS) were used to systematically characterize the TPA-CN (Figures S2–S4).

The photophysical properties of TPA-CN were first investigated. From the fluorescence spectra in a solid state, TPA-CN obviously showed a more red-shifted fluorescence emission than TPA-CHO (Figure S5). The fluorescence lifetime of TPA-CN was about 0.38 ns, and the quantum yield was about 1.21% (Figure S6). TPA-CN had two ultraviolet absorption bands, whether in pure ethanol (EtOH) or in 90% EtOH/water solutions (Figure S7). Its molar extinction coefficient was about 1.609 × 10^4^ (in EtOH, 382 nm). In addition, TPA-CN showed good stability in various organic solvents, demonstrating the potential for application as a fluorescence probe.

**Figure 1:**
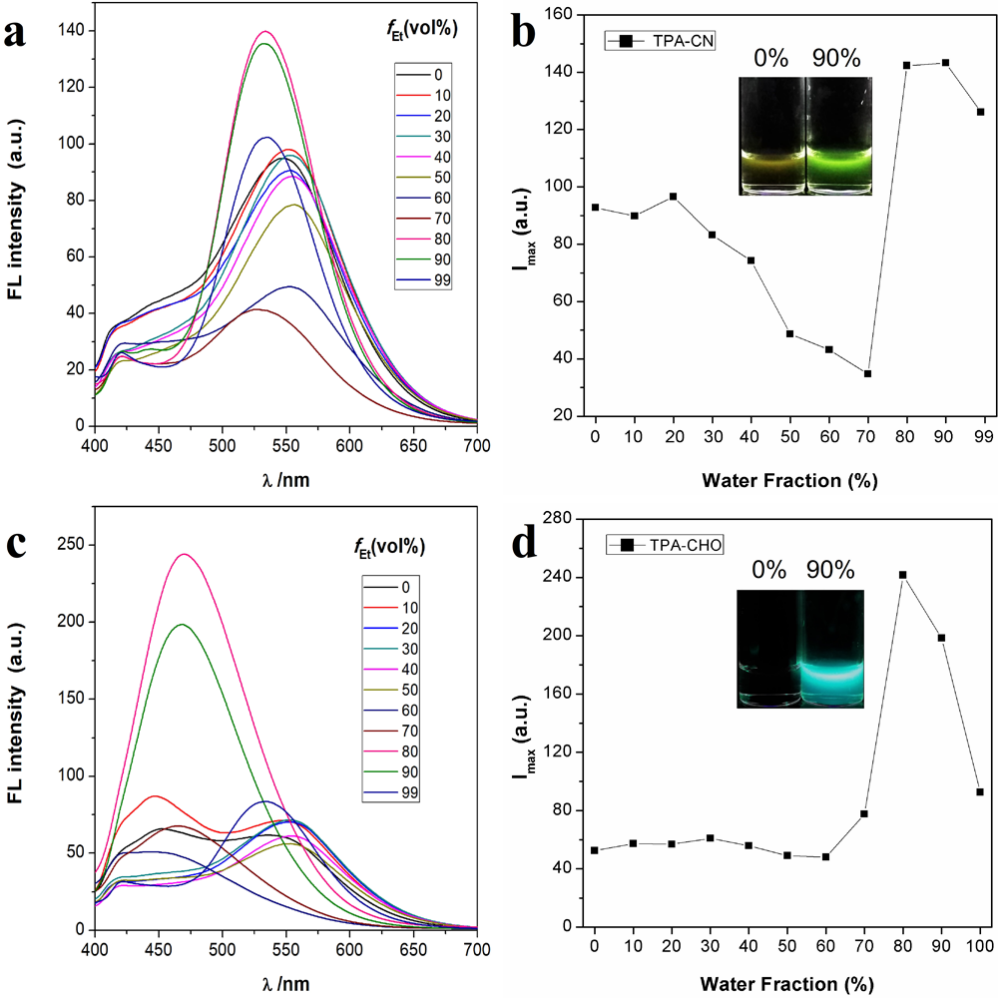
Photoluminescence (PL) spectra (**a**,**c**) and AIE curves (**b**,**d**) of TPA-CN (1 × 10^−4^ M) and TPA-CHO (1 × 10^−4^ M) in EtOH/water mixtures with different water fractions.

The photoluminescence properties of TPA-CN were investigated. The fluorescence spectra of TPA-CN showed polarity dependence, and the maximum fluorescence emission could range from ~470 nm to ~560 nm (Figure S8). The corresponding color could be changed from blue to yellow. By adjusting the fraction ratio of EtOH/water solutions, the aggregated and dissolved states of TPA-CHO and TPA-CN were controlled. The results showed that both TPA-CHO and TPA-CN exhibited classical AIE characteristics (Figure 1). For TPA-CN, the wavelength of maximum fluorescence emission was about 530 nm, which was consistent with the green emission, while the wavelength of maximum fluorescence emission was about 480 nm for TPA-CHO, and the emission color was cyan. Obviously, there was indeed a significant red shift in the fluorescence emission after the aldimine condensation reaction. The main reasons should be the increase in conjugation degree and the introduction of stronger electron-withdrawing groups. To further confirm the AIE properties of TPA-CN, the AIE curve in THF/water and DMSO/water mixed solutions are also investigated. The results showed similar trends as in the EtOH/water solutions (Figure S9). The average size of TPA-CN in 90% EtOH/water solutions was about 68.10 nm (Figure S10), demonstrating the enhanced fluorescence emission was really related to the aggregated state.

In addition to AIE properties, the TPA-CN also showed stronger twisted intramolecular charge transfer (TICT) characteristics [38,39]. This might be attributed to the long D-π-A structure in TPA-CN, which was much easier to twist in a polar environment. Firstly, a red-shifted fluorescence emission was observed during the increased polarity of the mixed solution. Once the aggregated state was formed at high water content larger than 70%, the molecular distortion was significantly suppressed, resulting in a blue-shifted emission and an increased luminescence intensity.

### 2.2. Acid Response Property of TPA-CN

**Figure 2:**
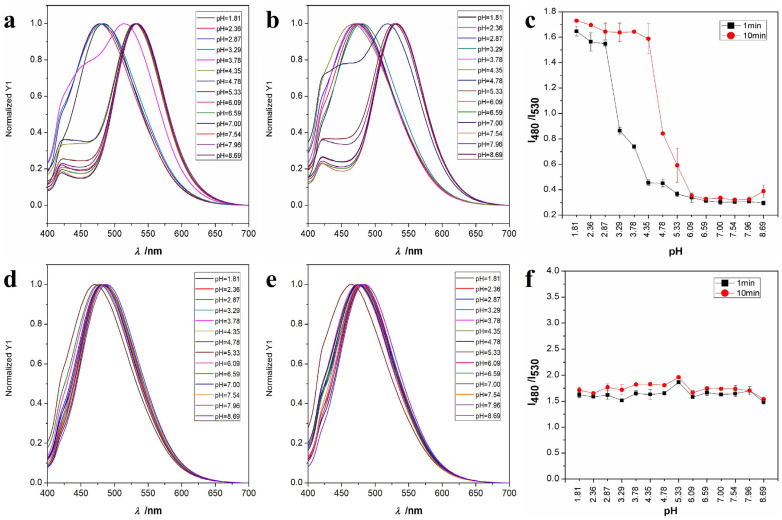
Normalized pH response of TPA-CN (5 μM) to BR buffers (**a**) for 1 min and (**b**) for 10 min. (**c**) Comparison of fluorescence ratios of TPA-CN to the pH response after 1 min and 10 min. Normalized pH response of TPA-CHO (5 μM) to BR buffers (**d**) for 1 min and (**e**) for 10 min. (**f**) Comparison of fluorescence ratios of TPA-CHO to the pH response after 1 min and 10 min.

BR buffer with different pH values (1.81, 2.36, 2.87, 3.29, 3.78, 4.35, 4.78, 5.33, 6.09, 6.59, 7.00, 7.54, 7.96, and 8.69) was used for the investigation of the acid response of TPA-CN (5 μM). For the group that was determined at 1 min, the obvious blue shift of fluorescence emission was observed in a pH lower than 3.78, and the intensity ratio (I_480_/I_530_) was about 0.74 ± 0.02. From the results determined at 10 min, the I_480_/I_530_ increased to 1.59 ± 0.12, and it began to stabilize at this value. The further extension of time no longer resulted in further changes, suggesting that pH 4.35 should be the critical response pH value of TPA-CN (Figure 2a–c). We also investigated the acid response property of TPA-CHO. There was no such obvious blue shift in the fluorescence emission, whether at 1 min or 10 min. The I_480_/I_530_ was maintained at about 1.52, demonstrating that the TPA-CHO was not sensitive to acid (Figure 2d–f).

Continuous monitoring of the fluorescence spectra within 10 min at different pH values (3.29, 4.35, and 5.33) was performed to further confirm the critical pH value of the TPA-CN. At pH 3.29 and 4.35, the wavelength of maximum fluorescence emission changed from 530 to 480 nm after an interaction of 10 min, and the I_480_/I_530_ was stable at 1.66 (Figure S11). It was worth noting that the I_480_/I_530_ at pH 4.35 went through a gradual process of change, which was much slower than the change process at pH 3.29, while at pH 5.33, the wavelength of maximum fluorescence emission was maintained at 530 nm, and the I_480_/I_530_ slightly increased to 0.42. Accordingly, pH 4.35 was further confirmed to be the critical pH value of the acid response. In such a case, the fluorescence emission information can help to confirm whether TPA-CN went through the acidic environment of the lysosome.

The reversibility of the blue-shifted fluorescence wavelength was investigated to reveal the mechanism of the acid response of TPA-CN. After the interaction of 10 min at pH 1.81 and 3.78, a significant blue shift of the fluorescence emission wavelength was observed as expected. However, there was no visible red shift of the fluorescence emission wavelength even if the pH value was adjusted back to 7.54 and through an interaction of 10 min (Figure S12). It can be concluded that there should be an irreversible reaction process accompanied by the blue-shifted fluorescence emission wavelength.

**Figure 3:**
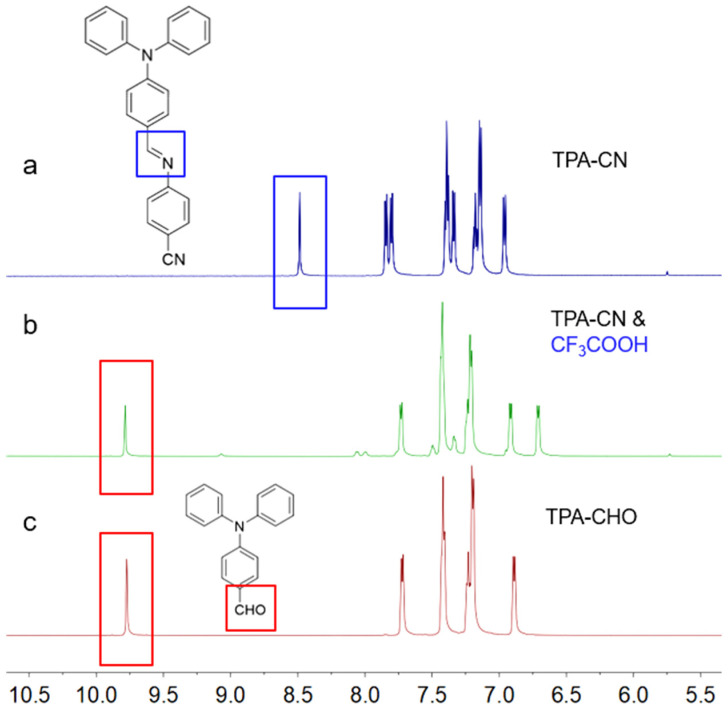
^1^H-NMR spectra investigation of the acid-response mechanism of the TPA-CN. (**a**) TPA-CN, (**b**) TPA-CN and CF_3_COOH, and (**c**) TPA-CHO. The key chemical shift peak and the corresponding functional group is noted by the same color box.

The ^1^H NMR spectra were used to further study the molecular mechanism during the acid response of TPA-CN. As shown in Figure 3, a classical chemical shift peak of the imine proton at 8.49 was observed in the ^1^H NMR spectra of TPA-CN (d-DMSO). After the treatment with trifluoroacetic acid, the chemical shift peak of the imine proton disappeared, and a new chemical shift peak at 9.78 emerged. This newly formed chemical shift peak can be attributed to the aldehyde group, which was consistent with the determined chemical shift peak of the aldehyde group (9.77) in TPA-CHO. Overall, the mechanism of the acid response of TPA-CN should be that the acid triggered the breakage of the imine bond, which led to the reduction in the conjugated structure and D-A effect and the accompanied blue-shift of the fluorescence emission wavelength.

### 2.3. LD-Targeting and Lysosomal Co-Localization Imaging

Before the cell imaging and LD-targeting imaging analysis, the cytotoxicity of TPA-CN was first investigated. The MTT assay results of 24 h cytotoxicity showed that the cytotoxicity was low when the concentration of TPA-CN was lower than 40 μM. The cell viability began to reduce to 82.55 ± 3.59 for 60 μM of TPA-CN, suggesting from this concentration that the TPA-CN began to exhibit a certain degree of cytotoxicity. For 80 and 100 μM of TPA-CN, the cell viability reduced to 66.59% ± 2.11 and 42.42% ± 2.63, revealing significant cytotoxicity (Figure S13). As a result, the concentration of TPA-CN should be controlled to be not higher than 40 μM in the following cell imaging and LD-targeting imaging.

**Figure 4:**
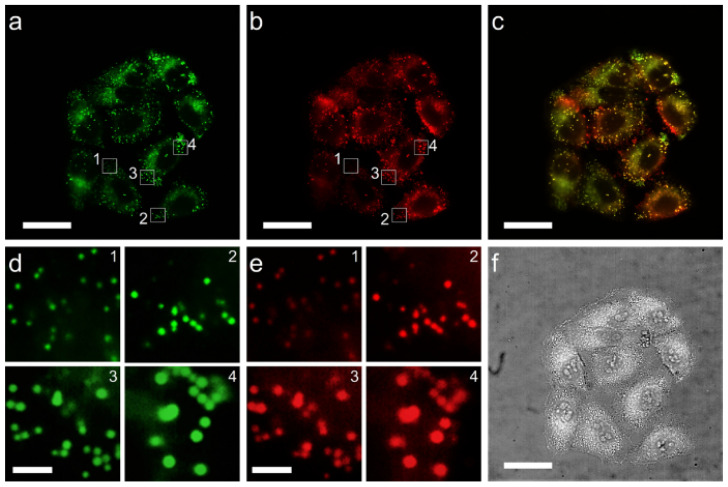
Lipid location fluorescence images of TPA-CN on HeLa cells. (**a**) The fluorescence imaging of the TPA-CN (20 μM) in Hela cells. (**b**) The fluorescence imaging of the NR (100 nM) in Hela cells. (**c**) The merged image of (**a**,**b**). (**d**,**e**) The enlarged images of LDs with different sizes (1–4) from the same places in (**a**,**b**). (**f**) The bright field image. Scale bar in (**d**,**e**) 2 μm and Scale bar in (**a**–**c**,**f**) 20 μm.

**Figure 5:**
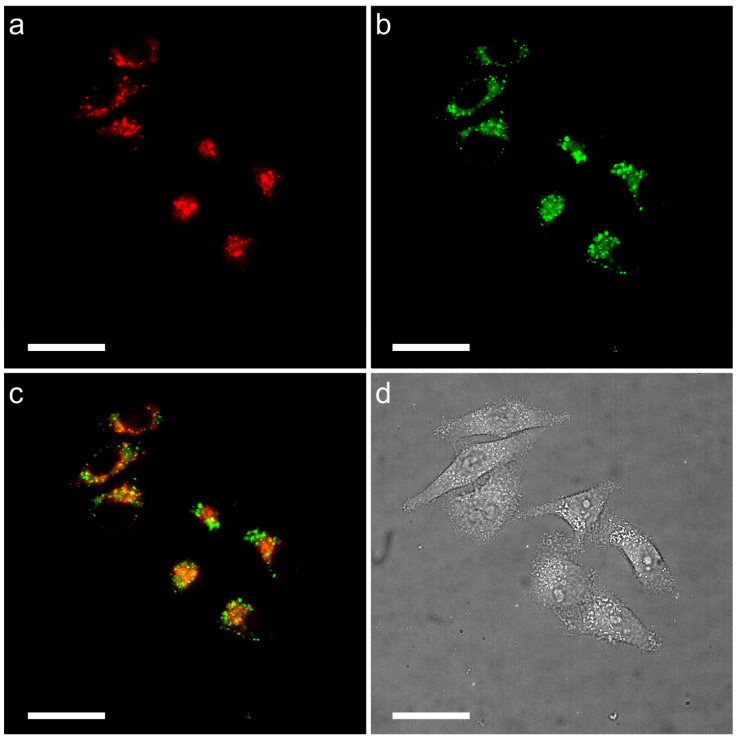
Fluorescence images of HeLa cells co-stained with TPA-CN and Lyso-Tracker Red. (**a**) Fluorescence images of HeLa cells with TPA-CN (20 μM). (**b**) Fluorescence images of HeLa cells with Lyso-Tracker Red (100 nM). (**c**) The emerged image of (**a**,**b**) the bight field image. (**d**) The bright field image. Scale bar 20 μm.

A confocal fluorescence imaging analysis system was used for cell imaging and the LD localization determination of TPA-CN. As shown in Figure 4, the green fluorescence of TPA-CN overlapped well with the red fluorescence of Nile red (NR), which was a widely used commercial LD dye. The fluorescence of TPA-CN was evenly distributed and had high brightness within the LDs. From the enlarged fluorescence image, it can be seen that TPA-CN was able to image LDs of different sizes with high sensitivity. The imaging effect of LDs with large particle sizes was comparable to that of NR, while the imaging visibility of LDs with small particle sizes even showed significant advantages (Figure 4d,e). The bright green fluorescence under the microscopy suggested the maintenance of the initial structure of TPA-CN. The co-localization imaging of TPA-CN with Lyso-Tracker Red was performed to further confirm whether the TPA-CN was distributed in the lysosome. The imaging results showed that no good overlap of the green fluorescence of TPA-CN and the red fluorescence of Lyso-Tracker Red was observed (Figure 5). Up to now, it can be concluded that the TPA-CN could target the LDs in HeLa cells, and during the internalization and LD-targeting process, its structure was stable. The lysosome pathway and lysosomal escape should not be involved in this process.

### 2.4. Dynamic Imaging of Intracellular LDs

As for the high signal-to-noise ratio of the LD-targeting imaging of TPA-CN, it was used to monitor the dynamic information of LDs. The dynamic process of the LDs stained with 20 μM TPA-CN was recorded within 10 min. By comparing the LD imaging at 0, 2, 4, 6, 8, and 10 min, it can be seen that the approximate position of the lipid droplets remained unchanged, and only a few lipid droplets experienced significant positional changes (Figure S14).

**Figure 6:**
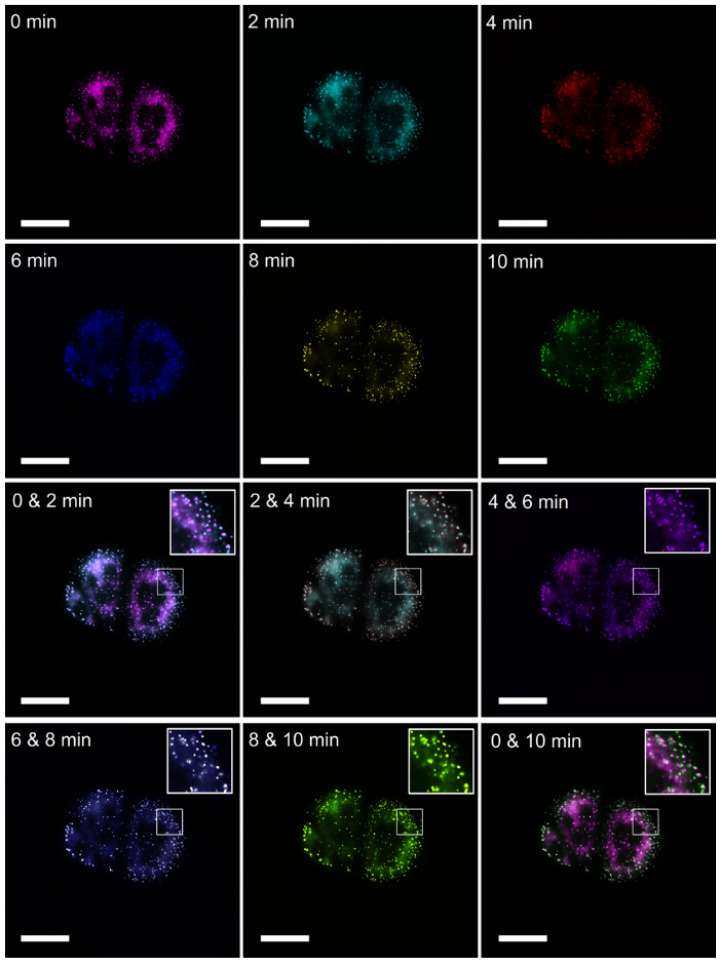
Contrastive analysis of LD location and dynamic monitoring images of TPA-CN (20 μM) on HeLa cells in 10 min. To better resolve the location and subtle change in the LDs, the imaging colors of LDs at different times were adjusted into different colors. The 0 and 2 min label means the merged image of 0 and 2 min, and the following are the same. Scale bar 20 μm.

To obtain a better overlay and resolution effect, the colors of the images at different times were changed to different colors (Figure 6). Then, the imaging results of 0 and 2 min, 2 and 4 min, 4 and 6 min, 6 and 8 min, 8 and 10 min, and 0 and 10 min were superimposed. There were no significant positional changes within a short time interval of 2 min, and there was no significant non-overlapping phenomenon in the previous five overlay maps. However, in the overlay maps of 0 and 10 min, it was evident that many lipid droplets had significant displacement, which was clear in the enlarged image. Obviously, the highly sensitive analysis of the subtle and slow positional information changes in LDs in HeLa cells was based on the high imaging visibility and signal-to-back ratio of TPA-CN, which also confirmed that TPA-CN can be used for highly sensitive imaging analysis and long-term imaging monitoring of intracellular LDs.

## 3. Materials and Methods

All chemicals and solvents used in the synthesis of TPA-CN and the imaging experiments were commercially purchased and directly used without further purification. NR was obtained from Aladdin Chemistry Co. Ltd. (Shanghai, China). Lyso-Tracker Red was purchased from Beyotime Biotechnology (Shanghai, China). ^1^H and ^13^C NMR were measured on Bruker Advance DMX 400 spectrophotometer (Bruker, Mannheim, Germany) using Chloroform-d (CDCl_3_) as solvent. During the mechanism investigation of the acid response property, d-DMSO was used as the solvent. High-resolution mass spectra (HRMS) were recorded on a GCT premier CAB048 mass spectrometer in the MALDI-TOF mode. The fluorescence spectra were recorded on a Hitachi F-7100 fluorescence spectrophotometer (Hitachi, Tokyo, Japan). The fluorescence lifetime and quantum yield were determined by an Edinburgh FLS 1000 fluorescence spectrophotometer (Edinburgh Instruments, Livingston, UK). The absorption spectra were measured by a Tianmei UV2310Ⅱ spectrophotometer (Shanghai Tian Mei Scientific Instrument Co., Ltd., Shanghai, China). Dynamic light scattering (DLS) measurements were carried out with a Malvern Nano-ZS Zetasizer. Confocal imaging was performed with Olympus IX81 microscopy. Fluorescence images of LDs were acquired using Image-Pro Plus Software (version 6.0). MTT assay was performed with Bio-Rad Model 680 microplate reader (Bio-Rad Laboratories, Hercules, CA, USA).

### 3.1. Synthesis of TPA-CN

TPA-CHO (2 mmol) and 4-Aminobenzonitrile (2 mmol) were dissolved into 20 mL of ethanol. After stirring at 50 °C for 24 h, the solution was cooled to room temperature. Finally, the light brown-yellow irregularly shaped crystals were collected through filtration, washed with ethanol several times, and used in the next step without further purification.

^1^H NMR (400 MHz, CDCl_3_) δ 8.29 (s, 1H), 7.69 (dd, *J* = 26.1, 8.4 Hz, 4H), 7.50–7.06 (m, 12H), and 7.06 (s, 2H). ^13^C NMR (400 MHz, CDCl_3_) δ 161.55 (s), 156.60 (s), 151.69 (s), 146.73 (s), 133.41 (s), 130.54 (s), 129.71 (s), 128.42 (s), 125.89 (s), 124.60 (s), 121.75 (s), 120.95 (s), 119.32(s), and 108.53 (s). MS *m*/*z* calcd for C_26_H_19_N_3_: [M + H]^+^ 374.1652, found 374.1628.

### 3.2. Acid Response Investigation of TPA-CN

Before the investigation of the pH responsiveness of TPA-CN, the speed of particle formation and the stability of fluorescence signals of TPA-CN in EtOH/water (1:99) solution were studied. According to the results, the fluorescence intensity began to reach maximum at 1 min and gradually stabilized; different pH buffer solutions were added and stabilized for 10 min before the fluorescence spectra were measured.

An amount of 5 μM of TPA-CN in Britton–Robinson (BR) buffer with different pH values was used for the investigation of the acid response property. Firstly, the fluorescence spectra of TPA-CN in EtOH/water (1:99) solution at 1 and 10 min were measured. Then, the change in the fluorescence intensities at pH 3.29, 4.35, and 5.33 were monitored within 10 min.

To further study the acid response mechanism, the reversibility of the acid-triggered blue-shifted wavelength was determined with fluorescence spectra. The fluorescence spectra of the TPA-CN in EtOH/water (1:99) solution at pH 3.78 and 1.81 for 10 min were first measured. Then, after the pH was adjusted back to neutral, the fluorescence spectra were measured again. In addition, the ^1^H NMR spectra of TPA-CN (d-DMSO) treated with trifluoroacetic acid were determined and compared with the TPA-CHO (d-DMSO).

### 3.3. In Vitro Cytotoxicity Assay

To evaluate the cytotoxicity of TPA-CN, 3-(4,5-dimethylthiazol-2-yl)-2,5-diphenyltetrazolium bromide (MTT) assays were performed on the HeLa cell line. For the MTT assay, logarithmically growing HeLa cells seeded in 96-well cell-culture plate at a density of 1 × 10^4^ cells per well were cultured in 5% CO_2_ at 37 °C overnight. Six compound holes were set up during the experiment. Then, fresh medium containing different concentrations of TPA-CN (2.5, 5, 10, 20, 40, 60, 80, and 100 μM) and the cells were further incubated for 24 h. Finally, the cell viability was assessed by MTT assay. An amount of 20 μL of MTT solutions were added into each hole. The culture medium containing MTT was discarded after an incubation of 4 h. Then, 150 μL of DMSO was added into each hole, followed by shaking for 10 min. The OD_490nm_ was determined to calculate the survival rate of cells. The percentage of cell viability compared to the untreated control cells was used to express the cytotoxicity.

### 3.4. Cell Imaging

Firstly, HeLa cells were used to investigate the LD-targeting ability of TPA-CN. An amount of 20 μM of TPA-CN was used to stain HeLa cells for 30 min. At 15 min, 100 μM of NR was added and incubated together with TPA-CN for 15 min. Then, the extra dyes were washed with PBS three times before the following fluorescence imaging analysis. A 60 × objective lens was used during the cell imaging.

To further confirm that the TPA-CN was not located at the lysosome, the co-stain experiments with TPA-CN (20 μM) and Lyso-Tracker Red (100 nM) for 30 min were performed. In addition, the HeLa cells stained with TPA-CN for 30 min were used for continuous and dynamic monitoring imaging of LDs. Before the dynamic monitoring imaging, the extra dyes were washed with PBS three times.

## 4. Conclusions

In conclusion, a multifunctional AIEgen TPA-CN, which contained a pH-response detachable connector, was designed for LD-specific imaging. The pH-response detachable connector made it responsive to the acidic microenvironment (pH < 4.35), making it responsive to the local acidic environment of the lysosome and exhibit an acid-triggered blue-shifted fluorescence emission. The TPA-CN showed LD-targeting imaging ability, and the internalization process did not involve the lysosome and the lysosomal escape process, which was based on its self-reporting ability. Furthermore, it was successfully used for the dynamic monitoring of the LDs with high resolution and better imaging ability than the commercial dye NR, especially for the stain of the small lipid droplets. This research might provide functional carriers that can avoid the lysosome route and accurately target the LDs.

## Data Availability

Not applicable.

## Supplementary Materials

The following supporting information can be downloaded at https://www.mdpi.com/article/10.3390/molecules28207029/s1. Figures S1–S10: characterization; Figures S11 and S12: pH-response investigation; Figure S13: cytotoxicity; Figure S14: cell imaging.

## Sample Availability

Samples of the compounds are not available from the authors.
