# Supplementary material for: A Multifunctional Aggregation-Induced Emission Luminogen with pH-Response Detachable Connector for Lipid Droplet-Specific Imaging and Tracing
Source: Molecules. 2023 Oct 11;28(20):7029. doi: 10.3390/molecules28207029 (PMC10608981; doi:10.3390/molecules28207029)
Supplement: Supplementary file 1 [file molecules-28-07029-s001.zip › molecules-2613766-supplementary.pdf]

Supporting Information

A Multifunctional Aggregation-Induced Emission Luminogen with pH-Response Detachable Connector for Lipid Droplet-Specific Imaging and Tracing

Yanjie Li 1, Rui Fan 2, Pengfei Gao 1,3,4,* and Chang-Hua Hu 1,4,*

1 College of Pharmaceutical Sciences, Southwest University, Chongqing 400715, China; lyj1989@swu.edu.cn

2 Southwest University Hospital, Southwest University, Chongqing 400715, China; fanrui01@swu.edu.cn

3 Key Laboratory of Luminescence Analysis and Molecular Sensing (Southwest University), Ministry of Education, Southwest University, Chongqing 400715, China

4 NMPA Key Laboratory for Quality Monitoring of Narcotic Drugs and Psychotropic Substance, Chongqing 401121, China

* Correspondence: gpf1987@swu.edu.cn (P.G.); chhhu@swu.edu.cn (C.-H.H.)

Supporting figures.

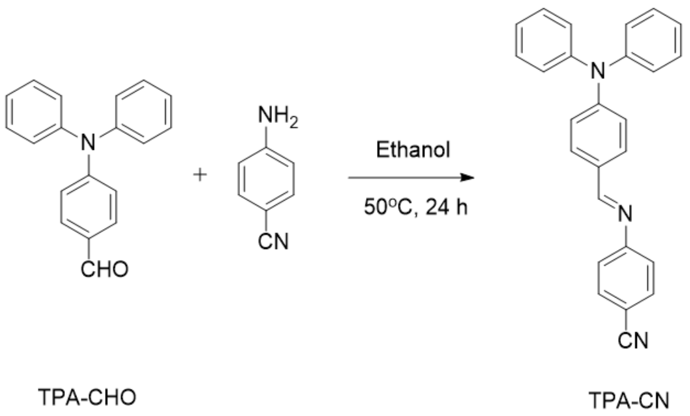

Figure S1 The synthesis route of Triphenylamine-benzonitrile Schiff base (TPA-CN) molecule.

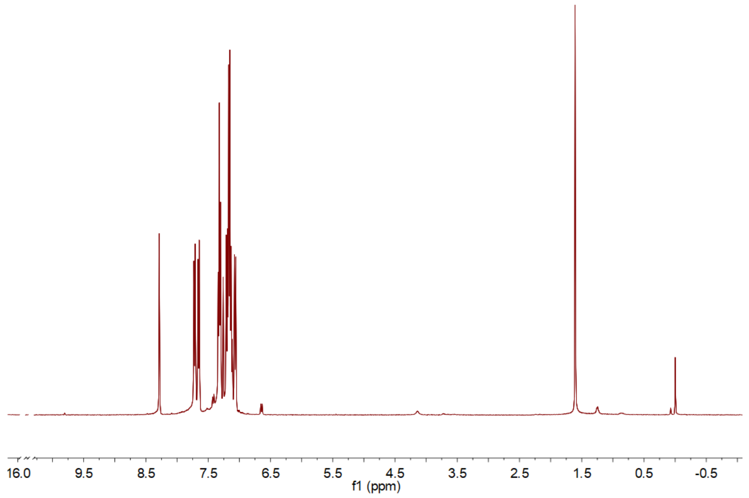

Figure S2 ^{1}H-NMR spectra of the TPA-CN.

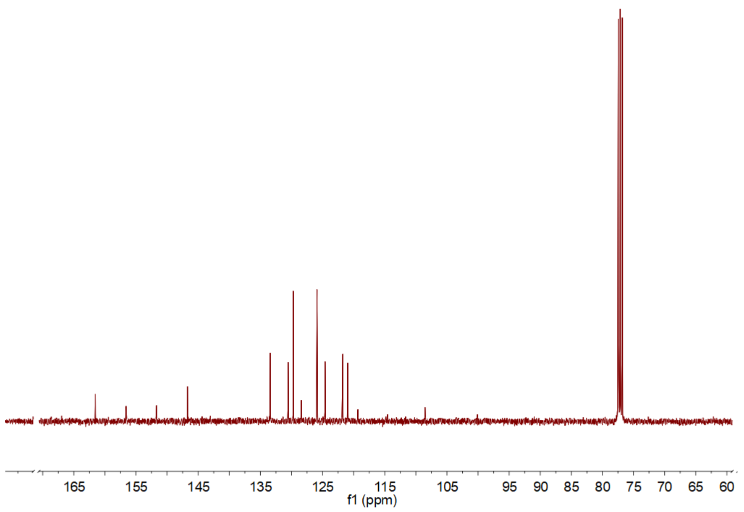

Figure S3 ^{13}C-NMR spectra of the TPA-CN.

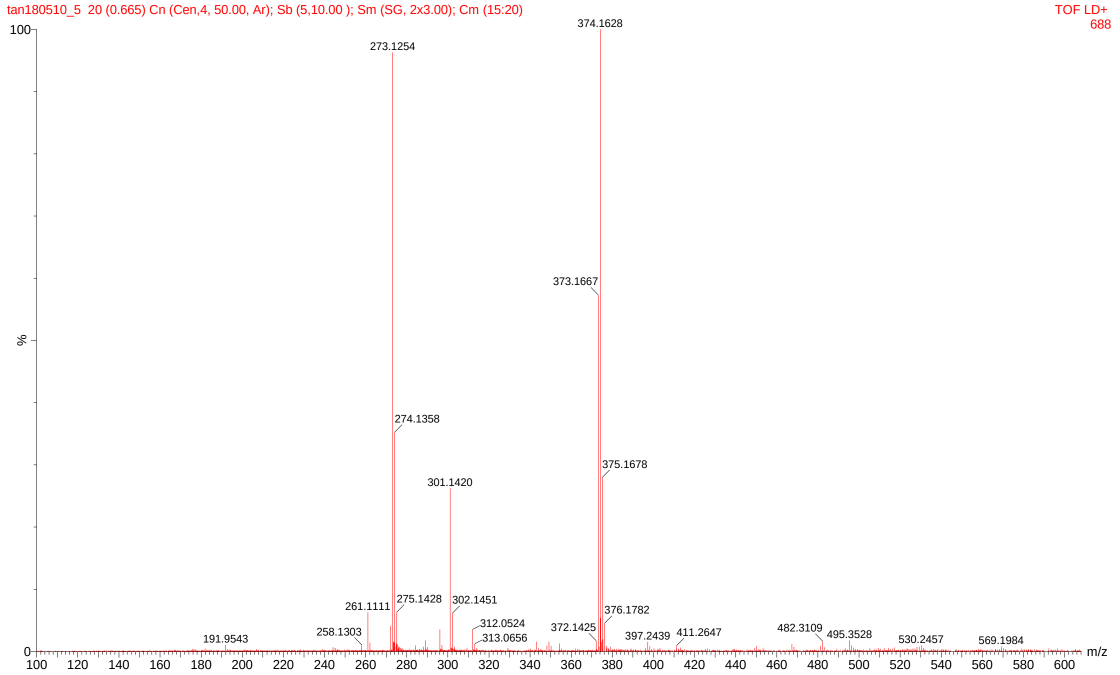

Figure S4 High resolution Mass spectra of the TPA-CN.

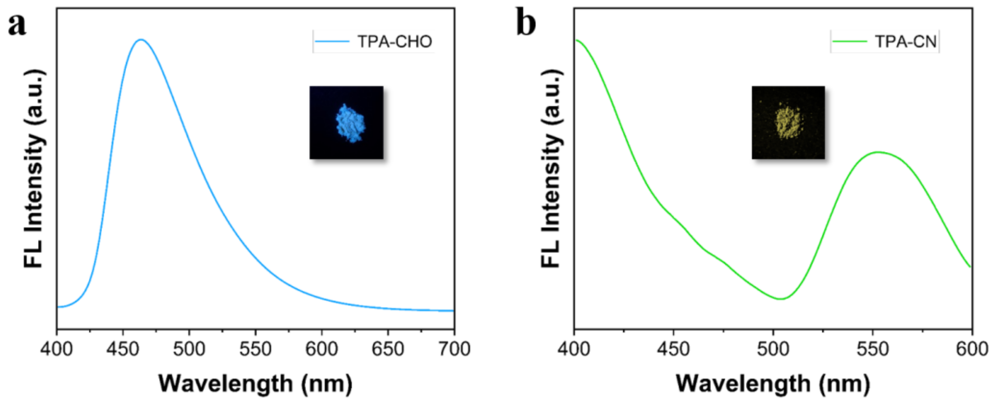

Figure S5 The fluorescence spectra of (a) TPA-CHO (λ_{ex} = 365 nm) and (b) TPA-CN (λ_{ex} = 330 nm). The inset graph was captured under UV irradiation of 365 nm.

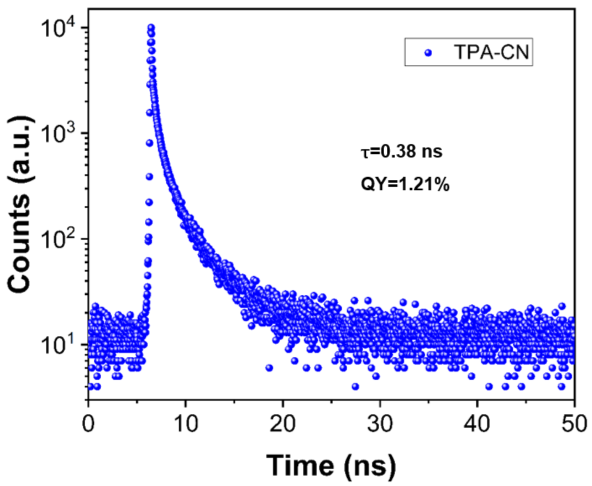

Figure S6 Fluorescence decay curves of the TPA-CN (λ_{em} = 550 nm).

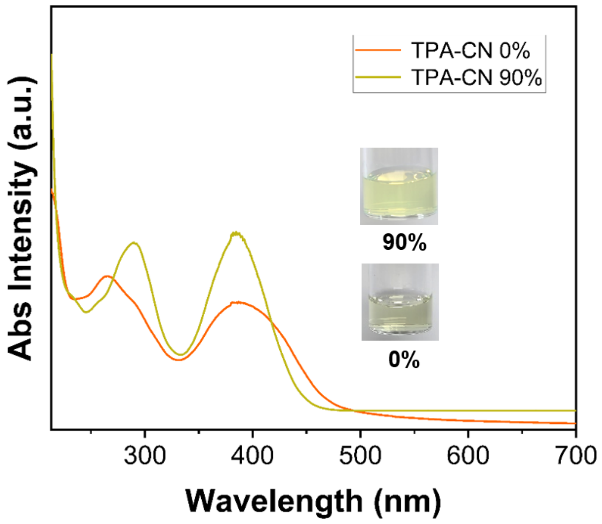

Figure S7 Absorption spectra of the TPA-CN (1×10^{-4} M) in pure ethanol and in 90% ethanol/water solution.

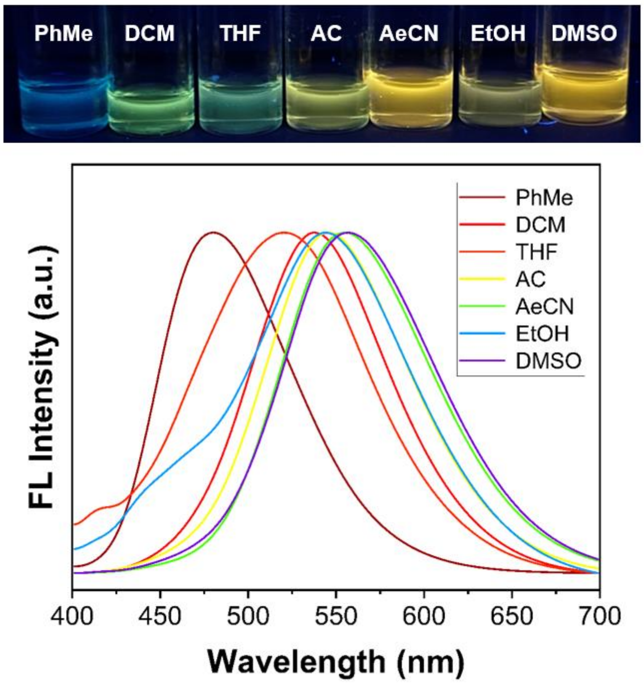

Figure S8 Fluorescence images and spectra of TPA-CN (1×10^{-4} M) in different solvents, including the toluene (PhMe), dichloromethane (DCM), tetrahydrofuran (THF), acetone (AC), acetonitrile (AeCN), ethanol (EtOH), dimethyl sulfoxide (DMSO), λ_{ex} = 365 nm.

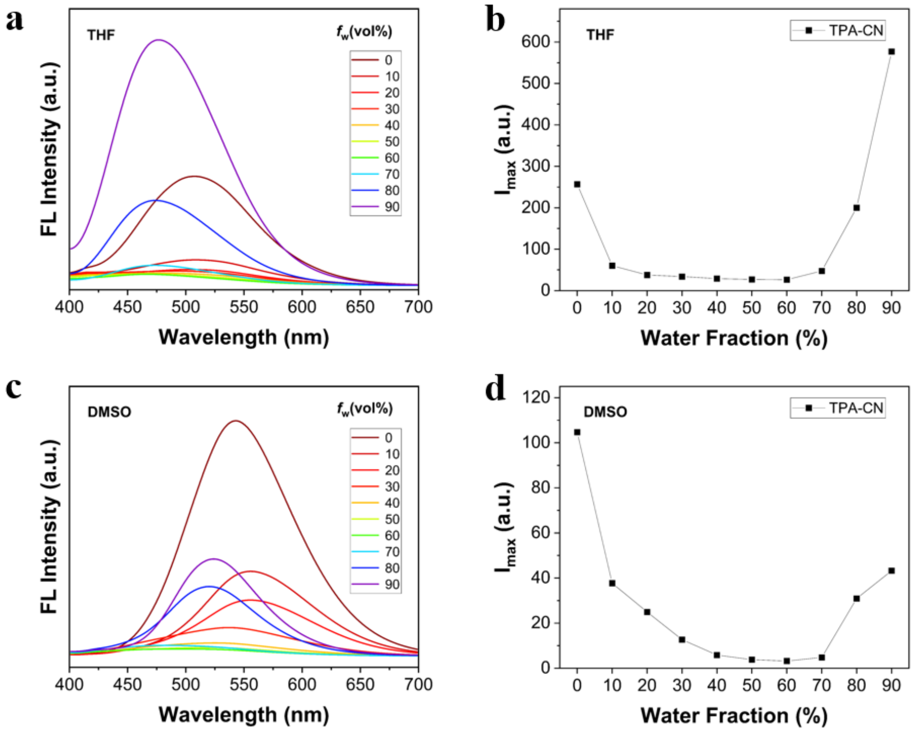

Figure S9 Fluorescence spectra (a, c) and AIE curves (b, d) of TPA-CN in THF/water and DMSO/water mixtures with different water fractions.

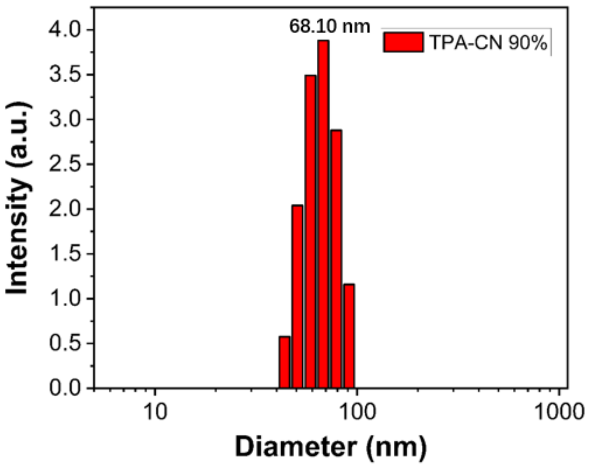

Figure S10 Size distribution profiles for the TPA-CN (1×10^{-4} M) in the 90% EtOH/water solutions determined by the DLS.

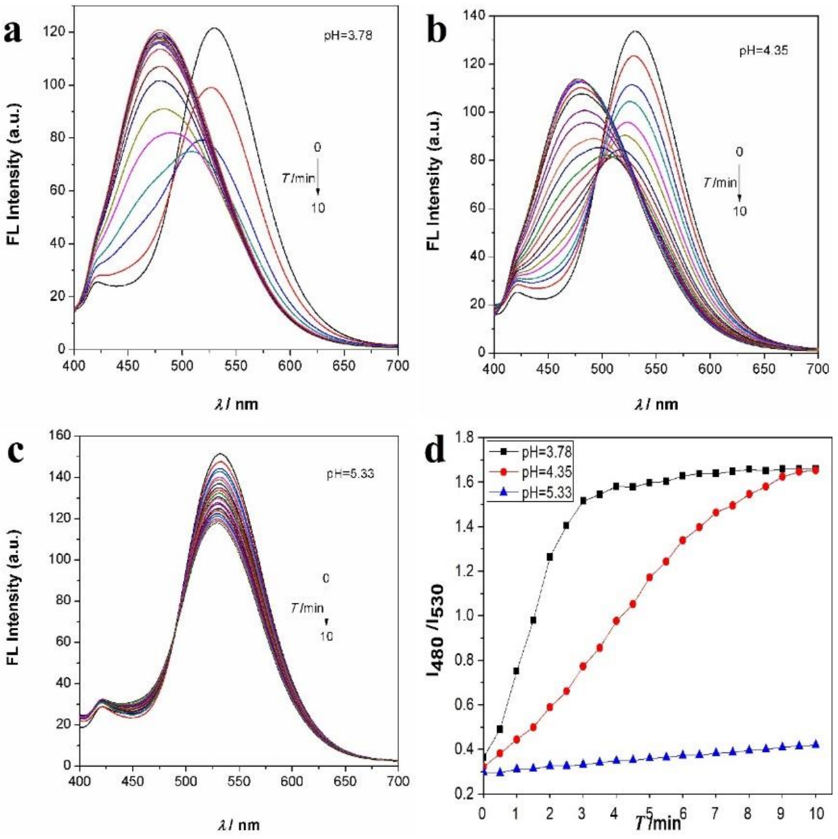

Figure S11 Photoluminescence (PL) spectra of TPA-CN in different pH BR buffers within 10 min. (a) pH response of TPA-CN to pH 3.29 BR buffers within 10 min. (b) pH response of TPA-CN to pH 4.35 BR buffers within 10 min. (c) pH response of TPA-CN to pH 5.33 BR buffers within 10 min. (d) Fluorescence ratios of TPA-CN within pH 3.29, 4.35 and 5.33 BR buffers within 10 min.

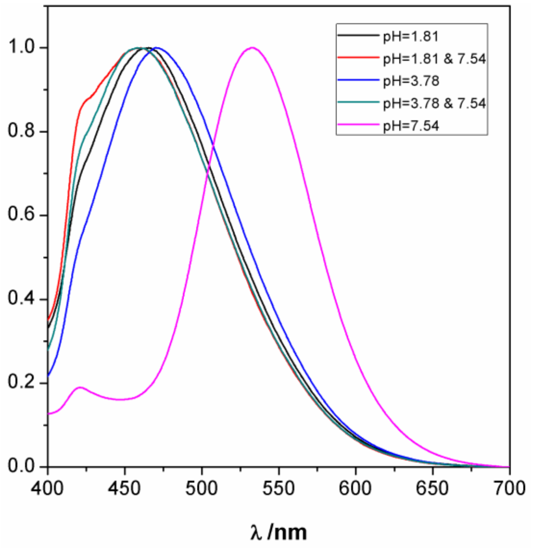

Figure S12 Investigation of the reversibility of the spectra response of TPA-CN to pH 1.81 and 3.78 BR buffers.

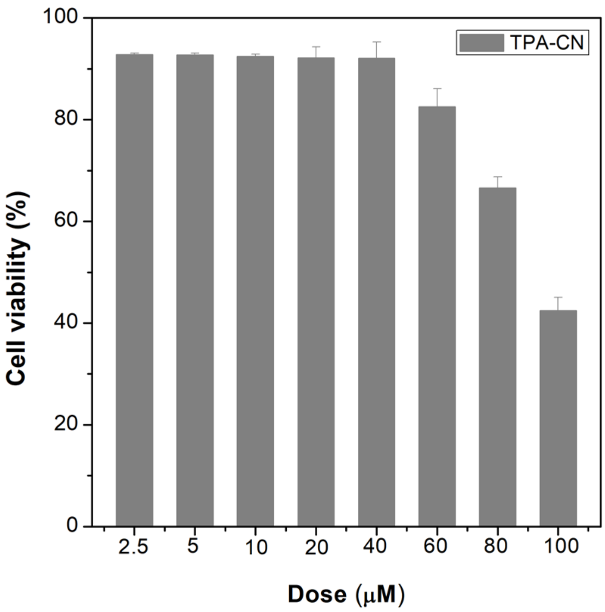

Figure S13 The cytotoxicity of TPA-CN on Hela cells. The concentration of TPA-CN was 2.5, 5, 10, 20, 40, 60, 80, 100 μM, and the time is 24 h.

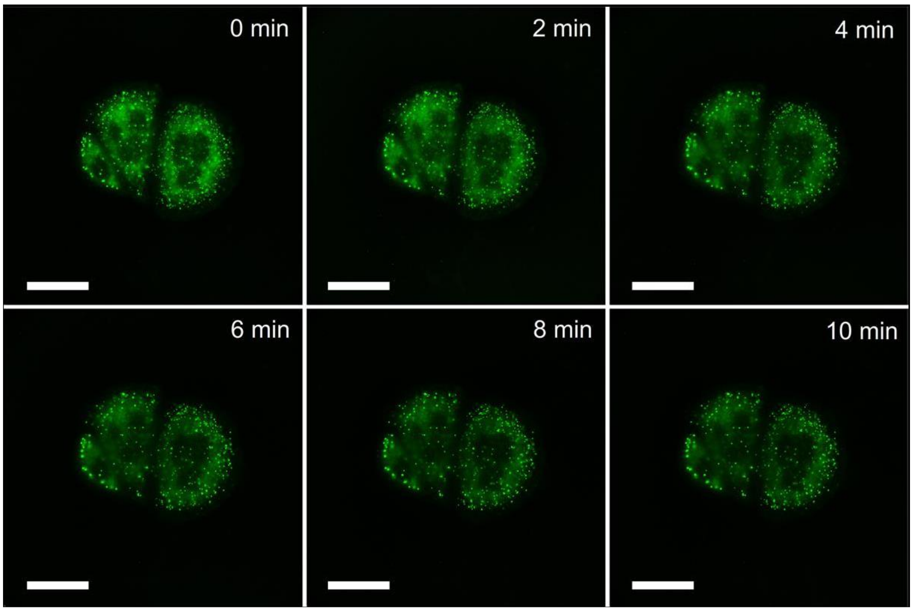

Figure S14 The original color of the contrastive analysis of LDs location and dynamic monitoring images of TPA-CN (20 μM) on HeLa cells in 10 minutes.
